# An Uncommon Culprit: Trapezius Dystonia as a Cause of Thoracic Outlet Syndrome: A Case Report

**DOI:** 10.7759/cureus.63825

**Published:** 2024-07-04

**Authors:** Jamie Philp, Won Jae Jeong, Paul Paily

**Affiliations:** 1 Physical Medicine and Rehabilitation, Baylor College of Medicine, Houston, USA

**Keywords:** trapezius hypertrophy, botulinum toxin injection, trapezius tightness, thoracic outlet syndrome, neurogenic thoracic outlet syndrome

## Abstract

Thoracic outlet syndrome (TOS) results from compression of the neurovascular bundle in the thoracic outlet. Several etiologies can contribute to the development of thoracic outlet syndrome, including both congenital and acquired causes. Historically, trapezius pathology has not been considered a cause of TOS; however, here we report a patient with neurogenic TOS plus ipsilateral trapezius hypertonicity and hypertrophy who had significant symptomatic improvement following botulinum toxin injections to trapezius.

## Introduction

Thoracic outlet syndrome (TOS) represents a group of disorders that arise due to compression of the neurovascular structures in the thoracic outlet. TOS can be classified as a neurogenic, arterial, or venous subtype depending on the compressed structure [1,2]. The etiologies of TOS are variable and can include congenital or acquired anatomic abnormalities, scalene or pectoralis muscle hypertrophy, and regional fibrosis due to trauma or repetitive arm motion [3].

Treatment of TOS typically involves a multidisciplinary approach tailored to the subtype and severity of symptoms. Conservative measures such as physical therapy focusing on posture correction, stretching, and strengthening exercises are often first-line [4,5]. In cases refractory to conservative management, surgical decompression may be considered. Although several studies show the benefit of surgical interventions in treating TOS, other studies support conservative management over surgical treatment due to its adverse effects or limited efficacy [6-11]. Alternatively, botulinum toxin injections to scalene, pectoralis minor, and subclavius have also been performed; this less invasive approach has shown promise in alleviating the symptoms of TOS, as temporary weakening of these muscles can help decompress the affected structures in the thoracic outlet [12-14].

Pathology of the trapezius muscle has not traditionally been considered as a cause of TOS as the trapezius does not directly border the thoracic outlet; however, the superior fibers of the trapezius attach to the distal clavicle, a recognized border of the thoracic outlet. We hypothesize that hypertonicity and hypertrophy of the trapezius contribute to the development of TOS through its attachment to the distal clavicle. Overactivity of the trapezius, particularly the superior fibers, can pull the distal clavicle superiorly and posteriorly, narrowing the costoclavicular space of the thoracic outlet. Here, we report a patient with neurogenic TOS plus ipsilateral trapezius hypertonicity and hypertrophy who had significant symptomatic improvement following botulinum toxin injections to trapezius. This supports the hypothesis that trapezius overactivity can contribute to the narrowing of the thoracic outlet and subsequent compression of the structures within.

## Case presentation

A 36-year-old female professional golf player with a history of multiple left shoulder labral repairs about a decade ago presented with three years of persistent left shoulder pain and reduced range of motion, and one year of numbness, tingling, and coldness in the first, fourth, and fifth digits of the left hand. Physical examination showed a reduced passive and active range of motion in her left shoulder, and an attempt at overhead activity resulted in pain in the trapezius, scalene, latissimus dorsi, and pectoral region. Given the possible comorbidity of adhesive capsulitis, the patient underwent several weeks of physical therapy, which significantly improved her shoulder range of motion. However, the patient continued to experience numbness, tingling, and coldness in her left digits, indicating a deeper underlying issue.

Upon further physical examination, the patient was noted to have significant hypertrophy and tightness of the transverse trapezius, tenderness in the scalene and pectoral areas, and decreased grip strength on the left side. Wright's test and Roos's test were positive, prompting suspicion of neurogenic thoracic outlet syndrome (NTOS). To exclude other possible diagnoses such as shoulder, cervical spine, or brachial plexus pathologies, various imaging, and interventions were performed: MRIs of the cervical spine and brachial plexus were unremarkable, and an intra-articular corticosteroid injection at the left shoulder provided no relief, suggesting a low likelihood of cervical spine, brachial plexus, or shoulder pathology and further supporting the diagnosis of NTOS. As a conservative therapeutic measure for NTOS, oral methocarbamol was prescribed, and the patient reported a mild but not dramatic improvement in her symptoms.

Given her history of professional golfing and the exam finding of tightness and hypertrophy of the left trapezius, we theorized that these trapezius abnormalities could be the culprits causing her NTOS. Although she also had tenderness in the scalene and pectoral areas, these muscles were not notably hypertonic compared to her trapezius. Therefore, we hypothesized that botulinum toxin injection to the trapezius may confer greater benefit than injection to the scalene or pectoralis muscles. Thus, we injected botulinum toxin into the superior fibers of her left trapezius. Following the first injection, the patient experienced a transient exacerbation of symptoms, which subsided after two weeks, later leading to improvement. A subsequent injection administered one year later resulted in marked improvement without any adverse effects. Overall, the patient reported a significant reduction of symptoms, with most days having minimal or no symptoms. The patient was able to return to her usual activities and is scheduled for a repeat botulinum toxin injection to her left trapezius.

## Discussion

The thoracic outlet is an anatomically complex region that spans from the supraclavicular fossa to the axilla. There are three areas where structures of the neurovascular bundle may be compressed within the thoracic outlet: the inter-scalene triangle (bordered by the anterior and middle scalene muscles and first rib), the costoclavicular space (bordered by the subclavius muscle, clavicle, and first rib), and the retro-pectoral space (bordered by pectoralis minor and ribs) [3]. The costoclavicular space is particularly relevant to our hypothesis because abnormal positioning of the clavicle can compress the brachial plexus in this area. The distal clavicle serves as the attachment point for the superior fibers of the trapezius, which originate from various points in the skull and neck and are inserted at the lateral third of the clavicle. When these fibers contract, they pull the clavicle superiorly and posteriorly, narrowing the costoclavicular space and exerting traction on the subclavius muscle. The subclavius, attaching the clavicle inferiorly to the first rib, contracts in response to counteract the trapezius's pull. This muscle action may explain the patient's tenderness in this region. Anatomically, the contractions of these muscles can cause functional disruption of the brachial plexus: the superior and posterior position of the clavicle compresses the brachial plexus posteriorly, while the subclavius's contraction elevates the first rib against the clavicle, further narrowing the costoclavicular space.

The diagnosis of NTOS is difficult as the differential diagnosis is broad, and there is no gold-standard diagnostic test. The differential diagnosis of NTOS includes peripheral nerve entrapments, cervical radiculopathy, brachial plexopathies, and various musculoskeletal conditions [15]. The diagnosis is usually suspected based on clinical presentation and specific risk factors. For example, in our patient’s case, the risk factors include her age, sex, history of multiple shoulder surgeries, and repetitive activity from golf. The diagnosis may also be supported by provocative physical exam maneuvers and radiographic findings, although radiographic findings may be normal. Over 90% of TOS cases are neurogenic TOS, with the lower brachial plexus most commonly compromised [3]. This patient’s risk factors, presentation, lack of response to intra-articular corticosteroid injection, and positive response to muscle relaxants supported the diagnosis of NTOS.

The first-line treatment for TOS is conservative and consists of physical therapy focused on stretching, weight control, and posture, as well as non-steroidal anti-inflammatory drugs (NSAIDs) and muscle relaxants. Antidepressants and anticonvulsants are also prescribed for neuropathic pain. Refractory cases are often managed with surgical decompression via first rib resection and/or scalenectomy [3]. The role of surgical intervention is debatable, however, as studies report mixed evidence regarding outcomes following surgical versus conservative management [6-11]. For example, a retrospective cohort performed by Orlando et al. (2015) reported over 90% rates of improvement or resolution of symptoms after first rib resection [6]. However, a study performed by Landry et al. (2001) reported no significant difference in functional outcome in patients who had first rib resection compared to conservative management over a mean follow-up period of 4.2 years [7], and Franklin et al. (2000) reported over 70% of patients who received surgery still had significant functional limitations over a mean follow-up period of 4.8 years [8]. Intraoperative complications of surgical management include pneumothorax, transient phrenic nerve injury, hematoma formation [9], and brachial plexus injury [10]. Patients may also require surgical revision due to tissue regrowth, inadequate resection, or scar tissue formation [16]. Thus, when the risks of surgery are weighed against potential benefits, surgical management of TOS remains controversial, particularly in patients with no clear anatomic abnormalities.

As an alternative to the surgical approach, botulinum toxin injection has shown promise as a less invasive therapeutic option for certain cases of TOS, particularly those with symptoms due to muscle overactivity [12-14]. By temporarily weakening the targeted muscles, botulinum toxin injection can help expand the space of the thoracic outlet, reducing compression of the neurovascular structures and alleviating symptoms [12]. This approach may be considered when conservative measures fail to provide adequate relief or in conjunction with other treatments in a comprehensive management plan for TOS. The target muscles previously described in the literature for botulinum toxin injection consist of scalene, pectoralis minor, and subclavius as they surround the thoracic outlet [13,17-19]. So far, injection of the trapezius muscle in treating TOS has never been described in literature except by Jordan et al. (2017); however, even in this study, the main targets were scalene, pectoralis minor, or subclavius muscles, and trapezius was only additionally targeted to treat pain and dystonia [14].

Although trapezius pathology has not been traditionally associated with the development of TOS, we observed a patient whose presentation was consistent with NTOS in the context of ipsilateral trapezius hypertonicity and hypertrophy. She had significant symptomatic improvement following two injections of botulinum toxin to the hypertrophied trapezius, suggesting that the pathology of this muscle contributed to her symptoms. We hypothesize that trapezius hypertonicity and subsequent hypertrophy may contribute to the development of TOS through its attachment at the distal clavicle. The superior and posterior positions of the clavicle, as well as the resulting activation of the subclavius elevating the first rib, together narrow the costoclavicular space and compress the structures within. Thus, as demonstrated here, patients with TOS and concurrent trapezius hypertrophy can benefit from botulinum toxin injections to trapezius. Trapezius hypertrophy may be underdiagnosed in patients with TOS as it is not a traditional target of treatment; therefore, patients with TOS should be evaluated for trapezius hypertonicity as a potential target of treatment. The reduction of trapezius hypertonicity (and subsequent reduction of hypertrophy) may provide symptomatic relief through physiologic decompression of this area.

## Conclusions

Thoracic outlet syndrome results from compression of the neurovascular bundle in the thoracic outlet. Patients with symptoms refractory to physical therapy and NSAIDs have been managed with botulinum toxin injections to the scalene and pectoralis muscles, as well as surgical decompression, with varying levels of success. Here, we report a patient with neurogenic TOS with marked trapezius hypertonicity and hypertrophy who had significant symptomatic improvement with botulinum toxin injections to the trapezius. Trapezius hypertrophy may contribute to the development of thoracic outlet syndrome by elevating the distal clavicle and narrowing the thoracic outlet. Botulinum toxin injection to the trapezius may help decompress the thoracic outlet and provide symptomatic relief as an effective nonoperative management strategy for TOS.
